# Vaccine design of coronavirus spike (S) glycoprotein in chicken: immunoinformatics and computational approaches

**DOI:** 10.1186/s41231-020-00063-0

**Published:** 2020-08-27

**Authors:** Eman A. Awadelkareem, Sumaia A. Ali

**Affiliations:** 1 Faculty of Veterinary Medicine, University of Khartoum, Khartoum, Sudan; 2 Department of Veterinary Medicine and Surgery, College of Veterinary Medicine, Sudan University of Science and Technology, Khartoum, Sudan

**Keywords:** IBV, Spike protein, B-and T-cell epitopes, Computational approaches, Vaccine design

## Abstract

**Background:**

Infectious bronchitis (IB) is a highly contagious respiratory disease in chickens and produces economic loss within the poultry industry. This disease is caused by a single stranded RNA virus belonging to Cronaviridae family. This study aimed to design a potential multi-epitopes vaccine against infectious bronchitis virus spike protein (S). Protein characterization was also performed for IBV spike protein.

**Methods:**

The present study used various tools in Immune Epitope Database (IEDB) to predict conserved B and T cell epitopes against IBV spike (S) protein that may perform a significant role in provoking the resistance response to IBV infection.

**Results:**

In B cell prediction methods, three epitopes (_*1139*_*KKSSYY*_*1144*_*,*_*1140*_*KSSYYT*_*1145*_*,*_*1141*_SSYYT_*1145*_) were selected as surface, linear and antigenic epitopes.

Many MHCI and MHCII epitopes were predicted for IBV S protein. Among them _982_YYITARDMY_990_ and _*983*_*YITARDMYM*_*991*_ epitopes displayed high antigenicity, no allergenicity and no toxicity as well as great linkage with MHCI and MHCII alleles. Moreover, docking analysis of MHCI epitopes produced strong binding affinity with BF_2_ alleles.

**Conclusion:**

Five conserved epitopes were expected from spike glycoprotein of IBV as the best B and T cell epitopes due to high antigenicity, no allergenicity and no toxicity. In addition, MHC epitopes showed great linkage with MHC alleles as well as strong interaction with BF2 alleles. These epitopes should be designed and incorporated and then tested as multi-epitope vaccine against IBV.

## Introduction

Infectious bronchitis virus (IBV) is a single Positive stranded RNA that belonging to coronavirus of the chicken (*Gallus gallus*). It’s a highly contagious respiratory disease in chickens that’s mainly severe for very young chicks. The signs of illness include tracheal rales, coughing, sneezing, nasal discharge and some strains may cause kidney damage [1, 2]. The disease can be transmitted in respiratory discharges and feces by infected chickens, and it is spread by aerosol, ingestion of contaminated feed and water, and contact with contaminated equipment or clothing. The virus is not transmitted via eggs [3]. The disease causes economic loss within the poultry industry, affecting the performance of meat-type and egg-laying birds. The disease can affect all ages, but the clinical disease is more severe in young chicks. Chicks become more resistant to IBV-induced mortality with the increasing age [4].

There are four structural proteins related to the envelope, the spike (S), membrane (M), envelope (E), and nucleocapsid (N) protein [5]. The spike ‘S’ glycoprotein which located at the surface of the virion. The membrane ‘M’ glycoprotein is partially exposed at the surface of the virion and also the nucleocapsid ‘N’ protein that located internally. The spike glycoprotein of IBV induces virus neutralizing (VN) and HI antibodies and has been considered as the most likely inducer of protection [2, 4]. The S protein is either a dimer or trimer. It has two recognized functions; binding the virus to receptor molecules on host cells, and activating fusion of the virion membrane with host cell membranes, releasing the viral genome into the cell [2]. The spike gene in particular the S1 part, is highly variable, due to insertions, deletions, substitutions and recombination events [6]. Application of vaccine is the most effective way to protect against pathogenic diseases, particularly when these pathogens have a high mortality rate such as IBV and viruses in general. On the other hand, the large number of serotypes and strains (genotype) of IBV specifically complicate control method. IBV has shift and drift property [7].

Inactivated and live-attenuated vaccines are employed to control the disease. However, inactivated vaccines often fail to induce strong cellular immunity, while live-attenuated vaccines can contribute to development of antigenic variant viruses [5]. The increasing number of new IBV serotypes, caused by frequent gene mutation and recombination, poses a major challenge for the prevention and control of infectious bronchitis disease [8].

RNA viruses such as IBV have high mutational rates. Thus, the most important step in the design of cross-protective peptide vaccine against IBV is to target the conserved epitopes of different IBV serotypes [5].

Presentation by MHC molecules is important for developing vaccinal immunity. MHC class I and class II molecules are typically highly polymorphic and polygenic [9]. Avian MHC class I and class II genes are localized into two regions (MHC-B and MHC-Y) on the chromosome 16. The MHC-B and MHC-Y haplotypes assort independently as the result of an intervening region that supports highly frequent recombination [9, 10]. Chicken MHC B–F molecules have been structurally and functionally related to mammalian MHC class I molecules and have been involved in the presentation of antigen to CD8 + T lymphocytes, which is important for antiviral immune response [11]. Recently, the design of epitope-based vaccines has been expanded by developments in genomics, proteomics and the understanding of pathogens. Epitope is the negligible immunogenic region of a sequence of proteins that specifically produces accurate immune responses [12]. The identification of specific B and T cell epitopes produced more desirable manipulation of immune response [13]. It is known that designing of multi-epitope vaccines using bioinformatics tools can significantly reduce the time and cost of production and produce satisfactory results [14, 15].

The production of safer and more reliable vaccines for controlling IBV is important. Therefore, the aim of this study is to analyze strains of spike (S) glycoprotein of infectious bronchitis virus reported in NCBI database using immunoinformatics and computational approaches to select all possible epitopes that can be used as multi-epitopes vaccine. Protein characterization was also achieved for IBV spike protein.

## Material and method

### Protein sequence retrieval

Spike (S) protein sequences of different infectious bronchitis virus (IBV) strains were retrieved from the GeneBank of National Central Biotechnology Information (NCBI) (http://www.ncbi.nlm.nih.gov/protein/) database in March 2019. The sequences were saved in FASTA format (Table 1).

**Table 1:** Accession numbers, date and area of collection of the retrieved sequences of Spike protein of IBV

No	Accession No	Country	Year	No	Accession No	Country	Year
1	**NP_040831.1**^**a**^	**UK**	**2018**	**47**	**AAV98206.1**	**USA**	**2002**
2	**AHX25911.1**	**China**	**2016**	**48**	**AVX27612.1**	**India**	**2004**
3	**AHX25902.1**	**China**	**2016**	**49**	**ALE71331.1**	**India**	**2018**
4	**AHX25893.1**	**China**	**2016**	**50**	**AJP16712.1**	**China**	**2015**
5	**AMK51938.1**	**China**	**2016**	**51**	**AJP16739.1**	**China**	**2015**
6	**AEP84746.1**	**China**	**2016**	**52**	**AFP50306.1**	**Korea**	**2015**
7	**AEP84736.1**	**China**	**2016**	**53**	**AFP50302.1**	**Korea**	**2012**
8	**ACX71849.1**	**China**	**2011**	**54**	**AFP50294.1**	**Korea**	**2012**
9	**ACX71844.1**	**China**	**2011**	**55**	**AFP50274.1**	**Korea**	**2012**
10	**ACX71842.1**	**China**	**2011**	**56**	**AEL12221.1**	**China**	**2012**
11	**AAU09490.1**	**China**	**2011**	**57**	**ADY62552.1**	**China**	**2012**
12	**AAY24433.1**	**Singapore**	**2005**	**58**	**ADV71785.1**	**Netherlands**	**2010**
13	**AAY24423.1**	**Singapore**	**2005**	**59**	**ACQ55230.1**	**Netherlands**	**2011**
14	**AAY21248.1**	**Singapore**	**2005**	**60**	**ARE67884.1**	**Pakistan**	**2017**
15	**AAY21247.1**	**Singapore**	**2005**	**61**	**ARB66180.1**	**China**	**2017**
16	**AAY21246.1**	**Singapore**	**2005**	**62**	**AQY55821.1**	**China**	**2017**
17	**AAY21245.1**	**Singapore**	**2005**	**63**	**AHX26172.1**	**China**	**2016**
18	**AAY21244.1**	**Singapore**	**2005**	**64**	**AHX26163.1**	**China**	**2016**
19	**AAY21243.1**	**Singapore**	**2005**	**65**	**AHX26154.1**	**China**	**2016**
20	**AAY21242.1**	**Singapore**	**2005**	**66**	**AHX26145.1**	**China**	**2016**
21	**AGW24533.1**	**India**	**2015**	**67**	**AHX26136.1**	**China**	**2016**
22	**AAW33786.1**	**USA**	**2006**	**68**	**AHX26127.1**	**China**	**2016**
23	**AER08740.1**	**Sweden**	**2012**	**69**	**AHX26118.1**	**China**	**2016**
24	**AER08739.1**	**Sweden**	**2012**	**70**	**AHX26109.1**	**China**	**2016**
25	**AER08729.1**	**Sweden**	**2012**	**71**	**AHX26073.1**	**China**	**2016**
26	**AER08728.1**	**Sweden**	**2012**	**72**	**AHX26064.1**	**China**	**2016**
27	**AER08727.1**	**Sweden**	**2012**	**73**	**AHX26055.1**	**China**	**2016**
28	**AER08726.1**	**Sweden**	**2012**	**74**	**AHX26046.1**	**China**	**2016**
29	**AER08725.1**	**Sweden**	**2012**	**75**	**AHX26037.1**	**China**	**2016**
30	**AER08724.1**	**Sweden**	**2012**	**76**	**AHX26028.1**	**China**	**2016**
31	**AER08723.1**	**Sweden**	**2012**	**77**	**AHX26019.1**	**China**	**2016**
32	**AER08722.1**	**Sweden**	**2012**	**78**	**AHX26010.1**	**China**	**2016**
33	**AER08721.1**	**Sweden**	**2012**	**79**	**AHX26001.1**	**China**	**2016**
34	**ADA83557.1**	**USA**	**2011**	**80**	**AHX25992.1**	**China**	**2016**
35	**ADA83467.1**	**USA**	**2011**	**81**	**AHX25983.1**	**China**	**2016**
36	**ABH01142.1**	**USA**	**2007**	**82**	**AHX25974.1**	**China**	**2016**
37	**ABH01141.1**	**USA**	**2007**	**83**	**AHX25965.1**	**China**	**2016**
38	**ABI26423.1**	**USA**	**2006**	**84**	**AHX25956.1**	**China**	**2016**
39	**AAK27168.1**	**China**	**2005**	**85**	**AHX25947.1**	**China**	**2016**
40	**ACH72794.1**	**China**	**2009**	**86**	**AHX25938.1**	**China**	**2016**
41	**AAW83034.1**	**China**	**2006**	**87**	**AHX25929.1**	**China**	**2016**
42	**ARS23139.1**	**Egypt**	**2014**	**88**	**ACJ50199.1**	**Singapore**	**2005**
43	**AHX25920.1**	**China**	**2016**	**89**	**ACO37566.1**	**Singapore**	**2005**
44	**ADP06504.1**	**USA**	**2012**	**90**	**AYG86360.1**	**SouthKorea**	**2018**
45	**AAA66578.1**	**UK**	**1995**	**91**	**AYG86347.1**	**SouthKorea**	**2018**
46	**AAA70235.1**	**USA**	**2002**	**92**	**AAV28722.1**	**China**	**2006**

^a^Refseq

### Structural analysis

Reference sequence of spike S protein (NP_040831.1) was analyzed to identify chemicals and physical properties including GRAVY (grand average of hydropathicity), half-life, molecular weight, stability index and amino acids atomic composition using an online tool Protparam [16]

Secondary structure of spike S protein of IBV was analyzed through PSIPRED [17]. The secondary structure of protein including helix, sheet, turn, and coil parameters was predicted using GOR IV server at https://npsa-prabi.ibcp.fr/cgi-bin/secpred_sopma.pl. TMHMM an online tool (http://www.cbs.dtu.dk/services/TMHMM/), used to examine the trans-membrane topology of S protein. Presence of disulphide-bonds were predicted through an online tool DIANNA v1.1. It makes prediction based on trained neural system [18]. CDD-BLAST (http://www.ncbi.nlm.nih.gov/BLAST/) [19–21] and PFAM (https://pfam.xfam.org/) [22] were used to search the defined conserved domains in the targeted protein sequences. Blastp in NCBI database (https://blast.ncbi.nlm.nih.gov/Blast.cgi) using reference sequence (refseq-protein) database was used to compare spike reference sequences of different coronaviruses in human and animals against IBV spike protein sequence. Phylogenetic tree was also constructed based on COBALT multiple alignment (https://www.ncbi.nlm.nih.gov/blast/treeview/treeView.cgi) [19, 20].

### Multiple sequence alignment and epitope conservancy assessment

The retrieved sequences of IBV S protein were aligned using Clustal program and consensus sequence was generated using the multiple sequence alignment (MSA) tool, Jalview version 2.10.5. (http://www.jalview.org/about/jalview-scientific-advisory-committee) [23]. Epitope conservancy analysis in Immune Epitope Database (IEDB) was used to detect potential epitope conservancy (http://tools.iedb.org/conservancy/) [24]. For calculating the conservancy score, the sequence identity threshold was kept at 80%.

### Phylogeny analysis

Phylogenetic tree of the retrieved sequences of spike (S) protein was performed using MEGA7.0.26 (7170509) software using maximum likelihood parameter [25].

### B cell prediction

The Immune Epitope Database (IEDB) (http://tools.iedb.org/mhci/) was used to predict B and T cell epitopes of IBV reference sequence of S protein (NP_040831.1) [26]. Linear B-cell epitopes were predicted using BepiPred from IEDB [27]. Emini surface accessibility prediction tool was used to predict surface located epitopes [28]. Whereas, the antigenic epitopes were investigated using kolaskar and Tongaonkar antigenicity method [29].

Discontinuous epitopes were predicted using DiscoTope server [30]. The parameter was set at ≥0.5 which indicated 90% specificity and 23% sensitivity. This method is based on surface accessibility and amino acid statistics in a collected form dataset of discontinuous epitopes found out by X-ray crystallography of antigen/antibody protein buildings. Chimera software was used to display the position of predicted epitopes clusters on 3D structure of S protein [31].

### T-cell epitope prediction

The T cell epitopes were predicted in human among different alleles of major histocompatibility complex class I (MHCI) and class II (MHCII).

MHC-I binding epitopes were predicted by the IEDB MHC I prediction tool at http://tools.iedb.org/mhci. The binding affinity of peptides to MHC I molecules was measured using artificial neural networks (ANN) method [32, 33]. Prior to prediction, peptide lengths were set as 9 mers. The half maximum inhibitory concentration (IC50) values needed for the binding of peptide to MHC-I molecules were set less than or equal to 300 nM.

The IEDB MHCII prediction tool was used for MHC class II molecules at (http://tools.iedb.org/mhcii/) [26]. Human MHC class II alleles (HLA DR, HLADP and HLADQ) were used for MHCII binding predication. The NN-align method was used with IC50 less or equal to 1000 nM [34].

### Antigenicity, allergenicity and toxicity of epitopes

VaxiJen v2.0 server was used to predict the antigenicity of the conserved regions (http://www.jenner.ac.uk/VaxiJen) [35]. The default prediction parameters and a threshold value of 0.4 were used. The in silico allergenicity prediction of epitopes was investigated using AllerTop v .2.0 (http://www.ddg-pharmfac.net/AllerTOP) [36]. While ToxiPred server was used to evaluate the toxicity of predicted epitopes. (http://crdd.osdd.net/raghava/toxinpred/) [37].

### Homology modeling

IBV reference sequence and the protein sequences of BF alleles (BF2 *2101 and BF2*0401) were submitted to Raptor X server (http://raptorx.uchicago.edu/) to design their three D structures [38–40]. PEPFOLD3 server was used for the homology modelling of MHCI epitopes (http://bioserv.rpbs.univ-paris-diderot.fr/services/PEP-FOLD3/) from amino acid sequences [41–43]. Chimera software 1.8 was used to visualize 3D structures of IBV spike S protein reference sequence and BF alleles [31].

### Molecular docking

To perform molecular docking, 3D structures of MHCI epitopes and BF alleles were submitted simultaneously to the PatchDock online autodock tools; an automatic server for molecular docking (https://bioinfo3d.cs.tau.ac.il/PatchDock/) [44]. The five top models were selected using firedock [44]. The results were visualized using the UCSF-Chimera software 1.8 [31].

## Results

### Structural analysis

The physiochemical properties of the spike S protein, measured through Protparam, showed that it contained 1162 amino acids (aa) with a molecular weight of 128,046.70 kDa. The spike protein showed an antigenic nature when subjected to Vaxijen v2.0.

Theoretical isoelectric point (PI) of spike protein was 7.71, indicating its positive in nature. An isoelectric point above 7 indicates the protein is charged positively. Near to 81 aa charges were found negative, whereas 84 aa found positive.

Protparam computed instability-index (II) 35.53, this categorize the protein as stable. Aliphatic-index 86.05, which devotes a thought to the proportional volume holding by aliphatic side chain and GRAVY value of the protein sequence is 0.012. Half-life of S protein shown as the total time taken for its vanishing after it has been synthesized in cell, computed as 30 h for mammalian-reticulocytes, > 20 h for yeast, > 10 h for *Escherichia coli*. The total numbers of Carbon (C), Oxygen (O), Nitrogen (N), Hydrogen (H) and Sulfur (S) were entitled by the formula: C_5737_H_8847_N_1495_O_1718_S_56_.

**Fig. 1:**
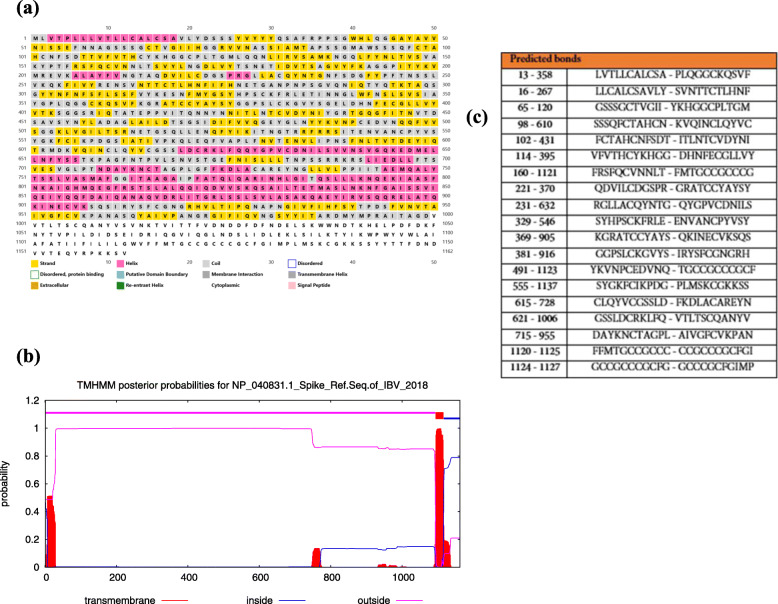
(**a**): The secondary structure of IBV spike protein; (**b**): transmembranr topology of spike protein; (**c**): the position of disulphides bond (S–S) in spike protein of IBV

The secondary structure of IBV spike S protein was analyzed through PSIPRED and GOR IV server. The components of secondary structure prediction by GOR IV server are alpha helix (29.43%), extended strand (27.37%), beta turn (5.25%), and random coil (37.95%) (Fig.1).

DiANNA1.1 tool calculated 19 disulphides bond (S–S) positions and assign them a score and makes prediction based on trained neural system. The trans-membrane protein topology was investigated via online tool TMHMM. Residues from 1 to 1093 were found to be exposed to the surface, residue from 1094 to 1116 were found inside trans-membrane-region and residues from 1117 to 1162 were buried within the core-region of the S protein (Fig.1).

**Fig. 2:**
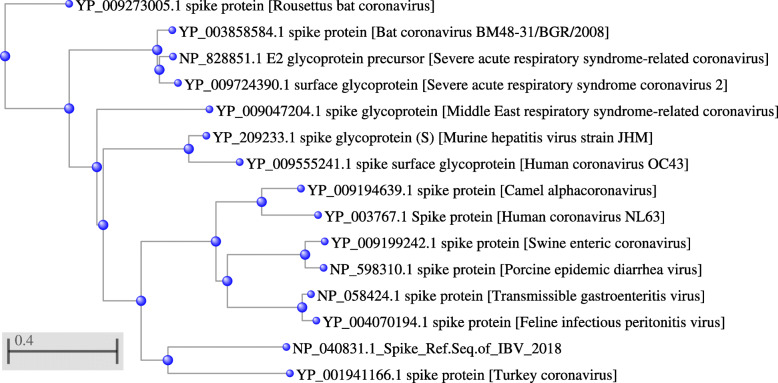
Phylogenetic tree of IBV against other coronaviruses in human and animals

In refseq of IBV spike protein two conserved domains (Corona-S2, Corona-S2) were identified. The conserved domains were sequenced by Conserved Domain (CDD) BLAST search. The results revealed that corona-S1 (pfam01600) is the only member of the superfamily cl03276 and corona-S2 domain (pfam01601) is the only member of the superfamily cl20218. The top associated sequences in both domains were Feline infectious peritonitis virus (strain 79–1146), Avian infectious bronchitis virus (strain Beaudette), and Human coronavirus 229E while Severe acute respiratory syndrome-related coronavirus sequences were associated only with corona-S2 domain. The closest homologue obtained from BLASTP (refseq-protein) results was the Turkey coronavirus S protein with E value 0.00 followed by Murine hepatitis virus strain JHM with E value 9e-109 when comparing various coronaviruses in human and animals with IBV spike protein sequence (Table 2). Phylogenetic tree of IBV against other coronaviruses in human and animals was created based on COBALT multiple alignment see Fig. 2.

**Table 2:** Blastp similarity search of IBV against other coronaviruses in human and animals

NCBI Protein ID	Protein Name	E- value	Identity
YP_001941166.1	Turkey coronavirus	0.0	38.59%
YP_009194639.1	Camel alphacoronavirus	8e-126	34.05%
YP_009199242.1	Swine enteric coronavirus	4e-124	31.47%
YP_003767.1	Human coronavirus NL63	2e-121	34.01%
NP_598310.1	Porcine epidemic diarrhea virus	8e-120	31.36%
YP_009273005.1	Rousettus bat coronavirus	1e-115	32.86%
NP_058424.1	Transmissible gastroenteritis virus	2e-109	32.03%
YP_209233.1	Murine hepatitis virus strain JHM	9e-109	37.20%
YP_004070194.1	Feline infectious peritonitis virus	1e-108	31.70%
YP_003858584.1	Bat coronavirus BM48–31/BGR/2008	4e-107	35.69%
NP_828851.1	E2 glycoprotein precursor [Severe acute respiratory syndrome-related coronavirus]	5e-107	36.28%
YP_009724390.1	Severe acute respiratory syndrome coronavirus 2	1e-106	36.31%
YP_009555241.1	Human coronavirus OC43	3e-105	31.42%
YP_009047204.1	Middle East respiratory syndrome-related coronavirus	2e-104	34.71%

### Multiple sequence alignment

**Fig. 3:**
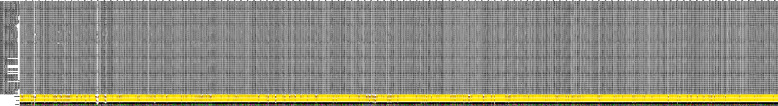
Multiple sequence alignment of spike (S) protein of IBV visualized by Jalview 2.10.5. *Yellow color bar and star sign indicate the full conservation*. The brown region indicates the mismatched sequences among them. Black bars show the consensus logo sequence and yellow color indicates good quality

Jalview was used to visualize the multiple sequence alignment of the retrieved sequences. Several areas in alignment were shown to have mutation see Fig. 3.

### Phylogeny

**Fig. 4:**
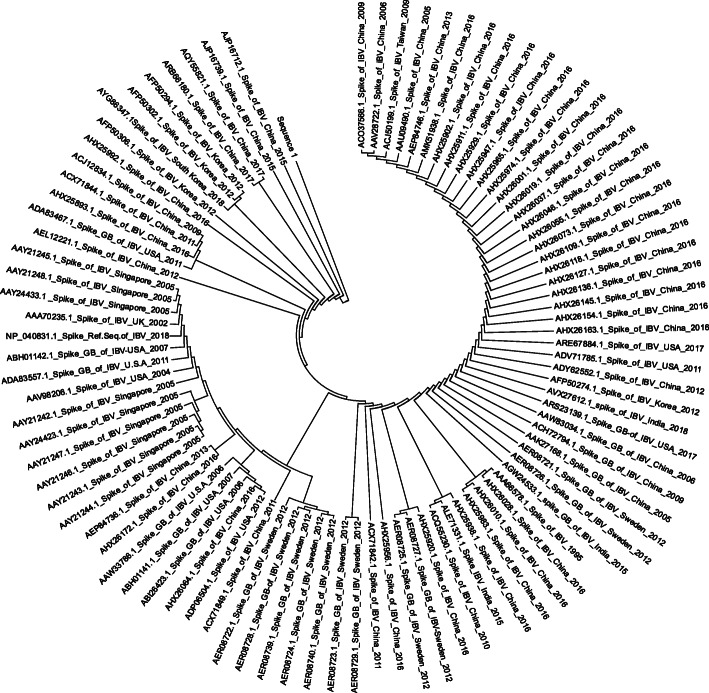
Phylogenetic tree of retrieved strains of spike protein using MEGA7.0.26 software

Phylogenetic tree for IBV spike S protein sequences was constructed using MEGA7.0.26 (7170509) software using maximum likelihood parameter see Fig. 4.

### B-cell epitopes

Several epitopes were predicted in B cell prediction methods using the Bepipred Linear Epitope Prediction tool. The conservancy percentages of these epitopes are presented in Table 3. After shortening of predicted epitopes, 21 linear conserved epitopes were recognized. Of these, seven epitopes with different lengths were identified as linear, surface and antigenic epitopes between the positions 1139–1146 (see Table 4). These epitopes were _*1139*_*KKSSYY*_*1144*_*,*_*1140*_*KSSYYT*_*1145*_*,*_*1141*_*SSYYTT*_*1146*_*,*_*1141*_*SSYYT*_*1145*_*,*_*1142*_*SYYTT*_*1146*_*,*_*1142*_*SYYT*_*1145,*_ and _*1143*_*YYTT*_*1146*_. Based on the length and antigenicity score, three epitopes (_*1139*_*KKSSYY*_*1144*_*,*_*1140*_*KSSYYT*_*1145*_*,*_*1141*_SSYYT_*1145*_) were selected as top B cell epitopes.

**Table 3:** Conservancy assessment of B cell linear epitopes

Epitope no	Epitope sequence	Start	End	Epitope length	Percent of protein sequence matches at identity <= 100%
1	MTAPSSGMAW	83	92	10	**89.13% (82/92)**
2	GGPI	193	196	4	**90.22% (83/92)**
3	TGNFSD	235	240	6	**97.83% (90/92)**
4	GPLQGGCK	352	359	8	**94.57% (87/92)**
5	DSAV	450	453	4	**91.30% (84/92)**
6	VNPCEDV	488	494	7	**96.74% (89/92)**
7	RNETGSQ	512	518	7	**94.57% (87/92)**
8	VGQKE	642	646	5	**81.52% (75/92)**
9	STKPAGFNTP	656	665	10	**81.52% (75/92)**
10	PQNAPN	926	931	6	**98.91% (91/92)**
11	ANASQY	959	964	6	**98.91% (91/92)**
12	IVPA	966	969	4	**86.96% (80/92)**
13	DFDFN	1026	1030	5	**84.78% (78/92)**
14	SKWWNDTKHELP	1034	1045	12	**94.57% (87/92)**
15	GKKSSYYTT	1138	1146	9	**97.83% (90/92)**

**Table 4:** List of shortened B cell epitopes predicted by different B cell scale

No.	Peptide	Start	End	Length	Emini	koleskar
1	MTAP	83	86	4	0.949	0.966
2	GSRIQT	406	411	6	1.273	0.973
3	SRIQT	407	411	5	1.583	0.992
4	SRIQ	407	410	4	1.355	1.013
5	STKP	656	659	4	2.543	0.979
6	VGLP	704	707	4	0.398	1.143
7	VGLPT	704	708	5	0.465	1.096
8	NASQY	960	964	5	2.034	1.006
9	SKWW	1034	1037	4	1.26	0.932
10	KKSSYYTT	1139	1146	8	6.723	1.003
11	KSSYYTT	1140	1146	7	4.166	1.013
12	SSYYTT^a^	1141	1146	6	2.568	1.027
13	SYYTT^a^	1142	1146	5	2.359	1.03
14	YYTT^a^	1143	1146	4	1.26	1.035
15	KKSSYYT	1139	1145	7	5.773	1.016
16	KKSSYY^a^	1139	1144	6	4.931	1.034
17	KSSYYT^a^	1140	1145	6	3.559	1.031
18	KKSSY	1139	1143	5	3.875	1.009
19	KKSS	1139	1142	4	3.054	0.971
20	SSYYT^a^	1141	1145	5	2.191	1.051
21	SYYT^a^	1142	1145	4	2.019	1.061

^a^Shortened peptide that has high score in both Emini and kolaskar

**Fig. 5:**
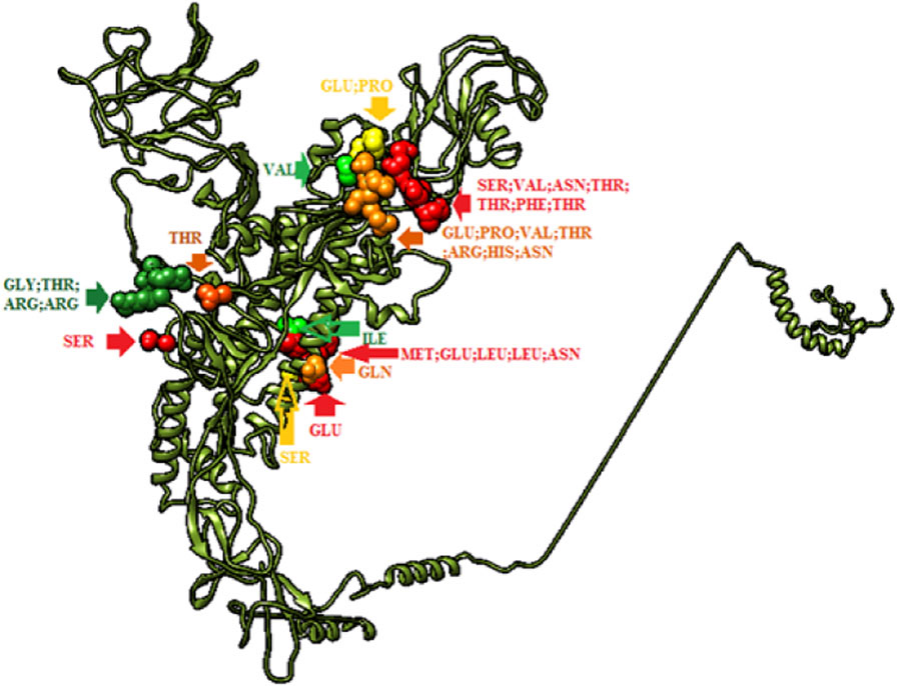
The position of proposed discontinuous B cell epitopes in the 3D structure of spike protein of IBV illustrated by UCSF-Chimera visualization tool

Discotope 2.0 server was used to predict the discontinuous epitopes from the 3D structure of S protein (PDB ID: 6CV0), 90% specificity, − 3.700 threshold and 22.000 Angstroms propensity score radius [45]. Total 30 discontinuous epitopes were recognized at different exposed surface areas (Table 5). The position of each predicted epitope on the surface of 3D structure of S protein is shown in Fig. 5 using Chimera visualization tool [31].

**Table 5:** Discontinuous epitopes predicted through DISCOTOPE 2.0 Server

Residue ID	Residue Name	Contact Number	Propensity Score	Discotope Score
262	SER	2	−3.91	−3.69
263	VAL	4	−2.626	−2.784
264	ASN	0	−0.238	−0.211
265	THR	19	−1.418	−3.44
266	THR	5	0.483	−0.148
267	PHE	25	−0.627	−3.43
268	THR	7	−0.463	−1.215
387	GLY	1	−3.678	−3.37
414	GLU	7	−0.476	−1.226
415	PRO	8	0.187	−0.754
417	VAL	5	−0.324	−0.862
419	THR	6	1.351	0.506
420	ARG	0	1.529	1.353
421	HIS	11	0.482	−0.838
422	ASN	12	−2.504	−3.596
515	THR	4	− 3.071	− 3.178
531	GLY	5	−1.472	− 1.877
532	THR	5	1.933	1.136
533	ARG	0	1.404	1.243
534	ARG	0	−0.425	−0.376
648	MET	5	−1.103	−1.551
649	GLU	16	−1.752	−3.39
650	LEU	26	−4.013	−6.541
651	LEU	10	−2.379	−3.256
652	ASN	12	−1.72	− 2.902
655	SER	7	−2.994	−3.454
685	SER	0	−3.842	−3.4
741	ILE	15	−1.303	−2.878
893	GLN	7	−2.411	−2.939
896	GLU	9	−2.901	−3.602

### Prediction of MHC class I epitopes

In this study, the Human MHC class-I HLA alleles were used to explore the interaction of epitopes with MHCI alleles as chicken MHC alleles don’t exists in IEDB database. MHC-1 binding prediction tool using IEDB database expected 13 conserved epitopes of spike protein (S) which were interacted with many cytotoxic T cell alleles. These epitopes were _*1115*_*FFMTGCCGC*_*1123*_*,*_*590*_*FNLTVTDEY*_*598*_*,*_*734*_*GLLVLPPII*_*742*_*,*_*1105*_*IIFILILGW*_*1113*_*,*_*1139*_*KKSSYYTTF*_*1147*_*,*_*1087*_*KTYIKWPWY*_*1095*_*,*_*166*_*SVYLNGDLV*_*174*_*,*_*985*_*TARDMYMPR*_*993*_*,*_*1145*_*TTFDNDVVT*_*1153*_*,*_*983*_*YITARDMYM*_*991*_*,*_*1144*_*YTTFDNDVV*_*1152*_*,*_*982*_*YYITARDMY*_*990*_*,*_*1143*_*YYTTFDNDV*_*1151*_.

### Prediction of MHC class II epitopes

MHC-II binding prediction tool based on NN-align with half-maximal inhibitory concentration (IC50) ≤ 1000 was used. Thirty one conserved core sequences were predicted to interact with MHCII alleles. These cores were _*694*_*EDLLFTSVE*_*702*_*,*_*1147*_*FDNDVVTEQ*_*1155*_*,*_*1115*_*FFMTGCCGC*_*1123*_*,*_*1116*_*FMTGCCGCC*_*1124*_*,*_*590*_*FNLTVTDEY*_*598*_*,*_*734*_*GLLVLPPII*_*742*_*,*_*1105*_*IIFILILGW*_*1113*_*,*_*902*_*INECVKSQS*_*910*_*,*_*984*_*ITARDMYMP*_*992*_*,*_*901*_*KINECVKSQ*_*909*_*,*_*1139*_*KKSSYYTTF*_*1147*_*,*_*1140*_*KSSYYTTFD*_*1148*_*,*_*1187*_*KTYIKWPWY*_*1195*_*,*_*735*_*LLVLPPIIT*_*743*_*,*_*592*_*LTVTDEYIQ*_*600*_*,*_*1014*_*NKTVITTFV*_*1022*_*,*_*893*_*QQRELATQK*_*901*_*,*_*894*_*QRELATQKI*_*902*_*,*_*895*_*RELATQKIN*_*903*_*,*_*589*_*SFNLTVTDE*_*597*_*,*_*1141*_*SSYYTTFDN*_*1149*_*,*_*166*_*SVYLNGDLV*_*174*_*,*_*1142*_*SYYTTFDND*_*1150*_*,*_*985*_*TARDMYMPR*_*993*_*,*_*1146*_*TFDNDVVTE*_*1154*_*,*_*593*_*TVTDEYIQT*_*601*_*,*_*1013*_*VNKTVITTF*_*1021*_*,*_*983*_*YITARDMYM*_*991*_*,*_*1144*_*YTTFDNDVV*_*1152*_*,*_*982*_*YYITARDMY*_*990*_ and _*1143*_*YYTTFDNDV*_*1151*_.

### Antigenicity, allergenicity and toxicity of MHCI and MHCII epitopes

**Fig. 6:**
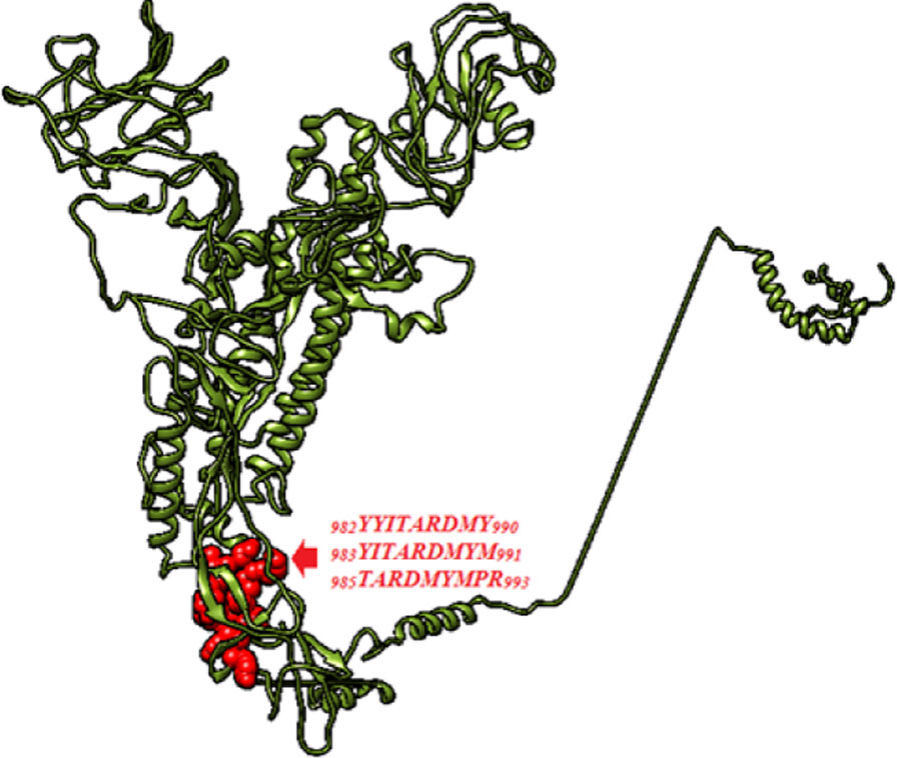
The position of proposed MHCI and MHCII epitopes (sphere red colour) in the 3D structure of spike protein of IBV illustrated by UCSF-Chimera visualization tool

The predicted epitopes of MHCI and MHCII were subjected to VaxiJen v2.0 server, AllerJen v2.0. and ToxiPred to estimate the potential antigenicity, allergenicity and toxicity of epitopes. Five MHCI epitopes were identified as antigenic, non-allergic and non-toxic, but only three epitopes (_*985*_*TARDMYMPR*_*993*_, _*983*_*YITARDMYM*_*991*_ and _*982*_*YYITARDMY*_*990*_) showed a high linkage with MHCI alleles (Table 6). Furthermore, six MHCII epitopes were predicted to be antigenic, non-allergic and non-toxic epitopes (Table 7). However, _9*83*_*YITARDMYM*_*991*_ and _*982*_*YYITARDMY*_*990*_ epitopes which were also presented in MHCII prediction methods, showed high antigenicity, no allergenicity and no toxicity. These epitopes were interacted with 52 and 38 alleles in MHCII see Fig. 6.

**Table 6:** Antigenic, non-allergic and non-toxic MHCI epitopes

Peptide	Start	End	Antigenicity	Allele	ic50
YYITARDMY	982	990	**0.8845**	HLA-A*29:02	14.52
				HLA-A*30:02	160.94
				HLA-C*14:02	27.32
YITARDMYM	983	991	**0.7901**	HLA-A*02:01	233.08
				HLA-A*02:06	212.86
				HLA-C*03:03	29
				HLA-C*06:02	200.39
				HLA-C*07:01	267.22
				HLA-C*14:02	49.52
				HLA-C*15:02	77.63
TARDMYMPR	985	993	**0.6914**	HLA-A*30:01	56.23
				HLA-A*31:01	14.3
				HLA-A*68:01	28.24
IIFILILGW	1105	1113	**0.6749**	HLA-B*57:01	78.45
				HLA-B*58:01	64.27
KKSSYYTTF	1139	1147	**1.1865**	HLA-A*32:01	182.52

**Table 7:** Antigenic, non-allergic and non-toxic MHCII epitopes

Core Sequence	Antigenicity	Peptide Sequence	Start	End	Allele	IC50
IIFILILGW	**0.6914**	IAFATIIFILILGWV	1100	1114	HLA-DRB1*15:01	454.6
KKSSYYTTF	**0.6749**	MSKCGKKSSYYTTFD	1134	1148	HLA-DPA1*01:03/DPB1*02:01	872.7
		SKCGKKSSYYTTFD	1135	1149	HLA-DPA1*01/DPB1*04:01	408.1
					HLA-DPA1*01:03/DPB1*02:01	301.5
					HLA-DPA1*02:01/DPB1*05:01	953.4
		KCGKKSSYYTTFDND	1136	1150	HLA-DPA1*01/DPB1*04:01	276.8
					HLA-DPA1*02:01/DPB1*05:01	853.9
		CGKKSSYYTTFDNDV	1137	1151	HLA-DPA1*02:01/DPB1*05:01	958.9
		MSKCGKKSSYYTTFD	1134	1148	HLA-DPA1*01:03/DPB1*02:01	872.7
KSSYYTTFD	**0.6466**	MSKCGKKSSYYTTFD	1134	1148	HLA-DRB1*04:05	155
		SKCGKKSSYYTTFDN	1135	1149	HLA-DRB1*04:05	125.6
		KCGKKSSYYTTFDND	1136	1150	HLA-DRB1*04:05	92.2
		CGKKSSYYTTFDNDV	1137	1151	HLA-DRB1*04:05	51.9
		GKKSSYYTTFDNDVV	1138	1152	HLA-DRB1*04:05	46.9
		KKSSYYTTFDNDVVT	1139	1153	HLA-DRB1*04:05	45.3
TARDMYMPR	**0.7901**	SYYITARDMYMPRAI	981	995	HLA-DRB1*03:01	269.3
		YYITARDMYMPRAIT	982	996	HLA-DRB1*03:01	281.9
		YITARDMYMPRAITA	983	997	HLA-DRB1*03:01	618.8
YITARDMYM	**1.1865**	QVNGSYYITARDMYM	977	991	HLA-DRB1*01:01	22
					HLA-DRB1*04:01	145
					HLA-DRB1*04:04	331.2
					HLA-DRB1*07:01	20.3
					HLA-DRB3*01:01	550.7
					HLA-DRB5*01:01	227.8
		VNGSYYITARDMYMP	978	992	HLA-DQA1*01:02/DQB1*06:02	338.6
					HLA-DRB1*01:01	25.8
					HLA-DRB1*03:01	447.6
					HLA-DRB1*04:01	105.8
					HLA-DRB1*04:04	248.3
					HLA-DRB1*07:01	27.8
					HLA-DRB1*15:01	380.6
					HLA-DRB3*01:01	577.8
					HLA-DRB5*01:01	198.6
		NGSYYITARDMYMPR	979	993	HLA-DQA1*01:02/DQB1*06:02	393.3
					HLA-DQA1*05:01/DQB1*03:01	817.3
					HLA-DRB1*01:01	19.8
					HLA-DRB1*03:01	176.5
					HLA-DRB1*04:01	65.2
					HLA-DRB1*04:04	225
					HLA-DRB1*07:01	40.2
					HLA-DRB1*15:01	291.2
					HLA-DRB3*01:01	635
					HLA-DRB5*01:01	93.5
		GSYYITARDMYMPRA	980	994	HLA-DQA1*01:02/DQB1*06:02	218
					HLA-DRB1*01:01	14
					HLA-DRB1*03:01	197.3
					HLA-DRB1*04:01	47.8
					HLA-DRB1*04:04	242.4
					HLA-DRB1*07:01	57.3
					HLA-DRB1*15:01	288.6
					HLA-DRB3*01:01	780.4
					HLA-DRB5*01:01	61.4
		SYYITARDMYMPRAI	981	995	HLA-DRB1*01:01	23.1
					HLA-DRB1*04:01	65.3
					HLA-DRB1*04:04	249.2
					HLA-DRB1*04:05	356.4
					HLA-DRB1*07:01	72.2
					HLA-DRB1*15:01	284.7
					HLA-DRB5*01:01	87.4
		YYITARDMYMPRAIT	982	996	HLA-DRB1*01:01	40.8
					HLA-DRB1*04:01	108.8
					HLA-DRB1*04:04	269.1
					HLA-DRB1*04:05	706.3
					HLA-DRB1*07:01	160.6
					HLA-DRB5*01:01	121.3
		YITARDMYMPRAITA	983	997	HLA-DRB1*04:01	145.4
					HLA-DRB1*04:04	652.4
					HLA-DRB1*07:01	355.4
					HLA-DRB1*08:02	955
					HLA-DRB5*01:01	206.9
YYITARDMY	**0.8845**	IQVNGSYYITARDMY	976	990	HLA-DQA1*05:01/DQB1*02:01	491.6
					HLA-DRB1*04:01	723.4
					HLA-DRB1*04:04	819.7
					HLA-DRB1*11:01	72
					HLA-DRB1*11:01	72
		QVNGSYYITARDMYM	977	991	HLA-DPA1*01/DPB1*04:01	710.8
					HLA-DPA1*01:03/DPB1*02:01	875.8
					HLA-DQA1*05:01/DQB1*02:01	292.7
					HLA-DRB1*03:01	588
					HLA-DRB1*11:01	32.4
					HLA-DRB1*11:01	32.4
					HLA-DPA1*01/DPB1*04:01	557.6
					HLA-DPA1*01:03/DPB1*02:01	860.8
					HLA-DQA1*05:01/DQB1*02:01	311.9
					HLA-DRB1*11:01	17.9
					HLA-DRB1*11:01	17.9
		NGSYYITARDMYMPR	979	993	HLA-DPA1*01/DPB1*04:01	503
					HLA-DPA1*01:03/DPB1*02:01	763.2
					HLA-DQA1*05:01/DQB1*02:01	387.6
					HLA-DRB1*09:01	858.7
					HLA-DRB1*09:01	858.7
					HLA-DRB1*11:01	11
					HLA-DRB1*11:01	11
		GSYYITARDMYMPRA	980	994	HLA-DPA1*01/DPB1*04:01	504.5
					HLA-DPA1*01:03/DPB1*02:01	790.4
					HLA-DQA1*05:01/DQB1*02:01	482.9
					HLA-DRB1*11:01	15.2
					HLA-DRB1*11:01	15.2
		SYYITARDMYMPRAI	981	995	HLA-DPA1*01/DPB1*04:01	480
					HLA-DPA1*01:03/DPB1*02:01	733.8
					HLA-DQA1*05:01/DQB1*02:01	526.3
					HLA-DRB1*11:01	26.8
					HLA-DRB1*11:01	26.8
		YYITARDMYMPRAIT	982	996	HLA-DPA1*01/DPB1*04:01	705.6
					HLA-DPA1*01:03/DPB1*02:01	931.5
					HLA-DQA1*05:01/DQB1*02:01	678.7
					HLA-DRB1*11:01	51.8
					HLA-DRB1*11:01	51.8

### Molecular docking

**Fig. 7:**
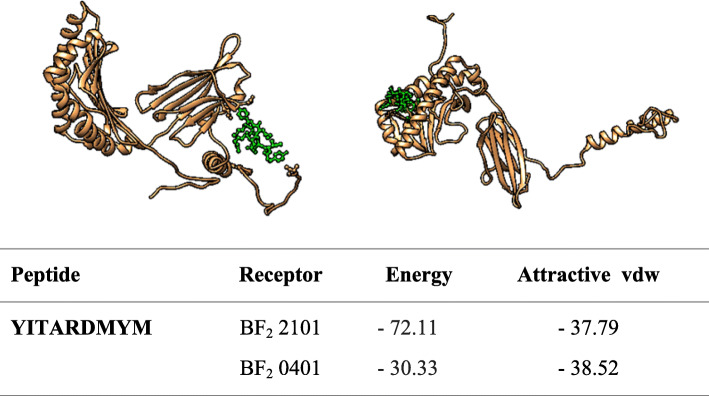
Docking of YITARDMYM with BF_2_ alleles

**Fig. 8:**
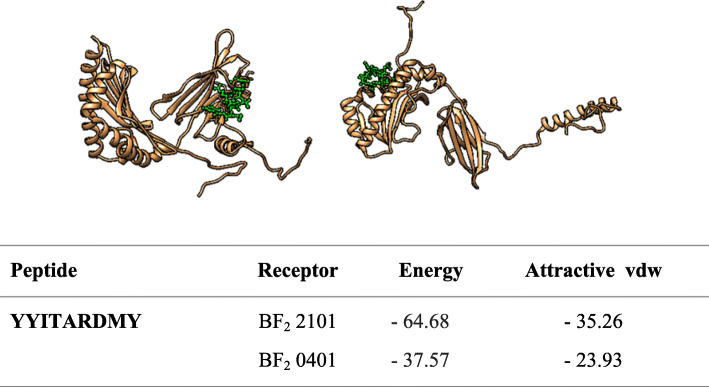
Docking of YYITARDMY with BF_2_ alleles

**Fig. 9:**
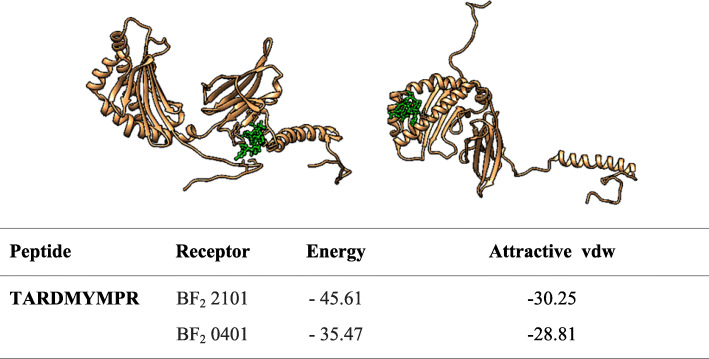
Docking of TARDMYMPR with BF_2_ alleles

The molecular docking was achieved by docking MHCI epitopes with chicken BF alleles (BF2 * 2101 & BF2 * 0401) using peptide-binding groove affinity. The chicken alleles were used as receptors, and the top MHCI epitopes _*982*_*YYITARDMY*_*990*_*,*_*983*_*YITARDMYM*_*991*_ and _*985*_*TARDMYMPR*_*993*_ were used as ligands. Docking of _*983*_*YITARDMYM*_*991*_ epitope with BF2*2101 and BF_2_*0401 alleles showed – 72.11 and – 37.39 global energy respectively, indicating a strong binding affinity between the ligands and the receptors compared to other epitopes (Fig. 7, 8 and 9). In general, the global binding affinity of ligands with the receptor BF2*2101 alleles was found to be lower compared to BF2*0401, suggesting strong receptor-ligand interaction.

## Discussion

Epitopes capable of inducing immunity in both types (B-cell and T-cell) are considered to be strong candidates for the vaccine [46]. There are several potential benefits offered by peptide vaccine over traditional vaccines against organisms. Most importantly, it allows the immune response to focus only on relevant epitopes and avoid those leading to non-protective responses, immune evasion, or unwanted side effects, such as autoimmunity [47].

IBV vaccination studies have always focused on humoral immune responses regarding protection. Acquired immunity results in the activation of antigen-specific effector mechanisms including B-cells (humoral), T-cells (cellular) and macrophages, and memory cells production [4]. Chickens develop a good humoral response to IBV infections, which measured by ELISA, virus neutralizing (VN) and haemagglutination-inhibition HI antibodies tests [48].

IBV glycoprotein S1 is known to be responsible for virus neutralization (VN) and haemagglutination-inhibition HI antibodies and has been considered the most likely protective inducer [4]. Multi-peptide vaccines using immunoinformatics tools have recently been conducted in Sudan for several viral diseases in chicken such as ILTV, fowlpox, Newcastle and marek’s disease virus [15, 49–51].

In the present study, IBV spike protein was analyzed using various prediction servers. Protein characterization of IBV spike S protein using Protparam confirmed its positive in nature and stable. The protein also exhibited good antigenic properties using Vaxijen 2.0v server.

Corona-S1 and Corona S2 have been identified as major conserved domains in the IBV spike glycoprotein refseq. Conserved Domain (CDD) BLAST search revealed that corona-S1 (pfam01600) is the only member of the superfamily cl03276 and corona-S2 domain (pfam01601) is the only member of the superfamily cl20218. The main related sequences in both domains were Feline infectious peritonitis virus (strain 79–1146), Avian infectious bronchitis virus (strain Beaudette), and Human coronavirus 229E. However, Severe acute respiratory syndrome-related coronavirus sequences was only associated with corona-S2 domain [52].

Prediction of B-cell epitopes is essential for the design vaccine components and immuno-diagnostic reagents. B-cell antigenic epitopes are either continuous or discontinuous in nature.

Most epitope prediction methods are based on continuous epitopes [53]. It has been reported that linear B cell epitopes play a role in virus neutralization [11]. IEDB prediction tool was used to predict linear, surface and antigenic epitopes based on the properties of amino acids such as hydrophilicity, surface accessibility, flexibility, and antigenicity [15].

In this study, seven shortened conserved epitopes (_*1139*_*KKSSYY*_*1144*_*,*_*1140*_*KSSYYT*_*1145*_*,*_*1141*_*SSYYTT*_*1146*_*,*_*1141*_*SSYYT*_*1145*_*,*_*1142*_*SYYTT*_*1146*_*,*_*1142*_*SYYT*_*1145,*_ and _*1143*_*YYTT*_*1146*_) were predicted from B cell prediction methods as surface, linear and antigenic epitopes. The predicted epitopes were adjacent to each other from the position 1139–1146. In a similar study, only one epitope (YTSNETTDVTS^175–185^) was predicted within the S1 glycoprotein of M41 IBV strains using BepiPred epitope prediction server version 1, and three such epitopes (VSNASPNSGGVD^279–290^, HPKCNFRPENI^328–338^, NETNNAGSVSDCTAGT^54–69^) were predicted in CR88 IBV strains [11].

The majority of B-cell epitopes are conformational (around a 90%) and only a minority of native antigens have linear B-cell epitopes [54]. Discotope server has been used for predicting discontinuous.

Epitopes from the 3D structure of the spike IBV reference sequence. Around 30 discontinuous epitopes with a specificity of 90% were recognized at different exposed surface areas. These epitopes have a significant advantage in identifying the native well-structured protein Ag [55].

Cytotoxic T lymphocytes (CTL) provide a critical arm of the immune system in eliminating autologous cells expressing foreign antigen. Unlike humoral immunity, the specificity of CTL activation depends on membrane receptors rather than secreted molecules, and antigen receptors of CTL interact with peptide determinants only in association with matched major histocompatibility complex (MHC) molecules. Virus-specific CTL have been shown to be important, if not critical, for resolution of infection and elimination of viral shedding [1].

It is stated that, the major histocompatibility complex MHC restricted CTL response can be associated with decreases in viral load, and CD8^+^ lymphocytes were mostly responsible for the observed protection [1, 56]. Responses to infectious bronchitis virus (IBV) with cytotoxic T-lymphocyte (CTL) were calculated at regular intervals between 3 and 30 days post infection [1].

However, MHCI prediction methods showed three conserved CTL epitopes _*985*_*TARDMYMPR*_*993*_, _*983*_*YITARDMYM*_*991*_ and _*982*_*YYITARDMY*_*990*_ as they linked to 7 and 3 human MHCI alleles respectively and showed high antigenicity, no allergenicity and no toxicity. Recent studies showed that vigorous cytotoxic T lymphocyte (CTL) responses that correlate with initial decrease in infection and illness can be detected after IBV infection. It has been identified that the CD8^+^ T cells were exhausted without CD4^+^ helper T cells. CD4^+^ T cells do not seem important in the initial resolution of IBV infection in chickens [56].

In MHCII prediction method, several core peptides were predicted to interact with MHCII alleles, but surprisingly the top core peptides were also _*983*_*YITARDMYM*_*991*_ and _*982*_*YYITARDMY*_*990*_ which were presented in MHCI prediction methods. They linked with 52 and 38 human alleles respectively. These epitopes showed high antigenicity, no allergincity and no toxicity.

Molecular docking was performed to display the interaction between BF alleles (BF2*2101 & BF2*0401) and MHCI epitopes (_*982*_*YYITARDMY*_*990*_*,*_*983*_*YITARDMYM*_*991*_ and _*985*_*TARDMYMPR*_*993*_). The 3D structures of MHC I epitopes were designed using PEPFOLD and docked with BF alleles via Patchdock server. Docking the epitope _*983*_*YITARDMYM*_*991*_ with both BF2 alleles produced strong binding affinity (− 72.11 and − 37.97 global energy respectively) followed by _*982*_*YYITARDMY*_*990*_ (− 64.68 and – 37.57 global energy respectively). This indicates the strong interaction between the ligand and the receptor compared to other epitopes (see Figs. 5, 6 and 7).

Ligands’ interaction with the receptor BF2*2101 alleles was found to be better compared with BF2 * 0401. However for both BF alleles, the docked molecules showed different groove binding site. Future studies should test the predicted epitopes for therapeutic potency to prove their safety and effectiveness**.**

## Conclusion

In this study, five epitopes were predicted from spike glycoprotein of IBV as the best B cell (_*1139*_*KKSSYY*_*1144*_*,*_*1140*_*KSSYYT*_*1145*_ and _*1141*_*SSYYT*_*1145*_) and T cell epitopes (_*982*_*YYITARDMY*_*990*_ and _*983*_*YITARDMYM*_*991*_). They showed high antigenicity, no allergenicity and no toxicity as well as great linkage of MHC epitopes with their alleles. The suggested epitopes should be designed, incorporated and tested as multi-epitopes vaccine against IBV. This vaccine may serve as a possible peptide vaccine to control IBV infection in chicken by inducing humoral and cellular responses.

Peptide vaccination against IBV spike protein (S) can strongly replace traditional vaccines as it is designed to cover all strains in different serotypes, which can reduce recurring outbreaks and their associated massive economic losses.

## Data Availability

All the data supporting the findings are contained within the manuscript.

## Abbreviations

IB: Infectious bronchitis
IBV: Infectious Bronchitis Virus
IEDB: Immune Epitope Database
S: Spike
MHC: Major histocompatibility complex
BF: The genetic polymorphism of properdin factor B
refseq: Reference sequence
NCBI: National Central Biotechnology Information
MSA: Multiple sequence alignment
GRAVY: Grand average of hydropathicity
CDD: Conserved Domain Database
IC50: The half maximal inhibitory concentration
ANN: Artificial neural networks
NN-align: Artificial neural network-based alignment
HLA: The human leukocyte antigen
CTL: Cytotoxic T lymphocytes
